# Web-Based Information on the Treatment of Tobacco Dependence for Oral Health Professionals: Analysis of English-Written Websites

**DOI:** 10.2196/jmir.8174

**Published:** 2017-10-20

**Authors:** Márcio Diniz-Freitas, Angel Insua, Ross Keat, Jean Christophe Fricain, Sylvain Catros, Luis Monteiro, Luis Silva, Giovanni Lodi, Alberto Pispero, Rui Albuquerque

**Affiliations:** ^1^ School of Medicine and Dentistry University of Santiago de Compostela Santiago de Compostela Spain; ^2^ Medical-Surgical Dentistry Research Group (OMEQUI) Health Research Institute of Santiago de Compostela (IDIS) University of Santiago de Compostela Santiago de Compostela Spain; ^3^ Birmingham Dental Hospital School of Dentistry University of Birmingham Birmingham United Kingdom; ^4^ Department of Dentistry University of Bordeaux Bordeaux France; ^5^ Department of Medicine and Oral Surgery University Institute of Health Sciences (IUCS) CESPU Gandra Portugal; ^6^ Institute of Research and Advanced Training in Health Sciences and Technologies (IINFACTS) CESPU Gandra Portugal; ^7^ Dipartimento di Scienze Biomediche Chirurgiche e Odontoiatriche University of Milan Milan Italy

**Keywords:** tobacco use cessation, Internet, general practice, dentistry, education, continuing

## Abstract

**Background:**

Studies have been conducted on the content and quality of Web-based information for patients who are interested in smoking cessation advice and for health care practitioners regarding the content of e-learning programs about tobacco cessation. However, to the best of our knowledge, there is no such information about the quality of Web-based learning resources regarding smoking cessation dedicated to oral health professionals.

**Objective:**

The aim of this study was to identify and evaluate the quality of the content of webpages providing information about smoking cessation for oral health care professionals.

**Methods:**

Websites were identified using Google and Health on Net (HON) search engines using the terms: smoking cessation OR quit smoking OR stop smoking OR 3As OR 5As OR tobacco counselling AND dentistry OR dental clinic OR dentist OR dental hygienist OR oral health professionals. The first 100 consecutive results of the 2 search engines were considered for the study. Quality assessment was rated using the DISCERN questionnaire, the Journal of the American Medical Association (JAMA) benchmarks, and the HON seal. In addition, smoking cessation content on each site was assessed using an abbreviated version of the Smoke Treatment Scale (STS-C) and the Smoking Treatment Scale-Rating (STS-R). To assess legibility of the selected websites, the Flesch Reading Ease (FRES) and the Flesch-Kinkaid Reading Grade Level (FKRGL) were used. Websites were also classified into multimedia and nonmultimedia and friendly and nonfriendly usability.

**Results:**

Of the first 200 sites selected (100 of Google and 100 of HON), only 11 met the inclusion criteria and mainly belonged to governmental institutions (n=8), with the others being prepared by Professional Associations (n=2) and nonprofit organizations (n=1). Only 3 were exclusively dedicated to smoking cessation. The average score obtained with the DISCERN was 3.0, and the average score in the FKRGL and FRES was 13.31 (standard deviation, SD 3.34) and 40.73 (SD 15.46), respectively. Of the 11 websites evaluated, none achieved all the four JAMA benchmarks. The mean score of STS-R among all the websites was 2.81 (SD 0.95) out of 5. A significant strong positive correlation was obtained between the DISCERN mean values and the STS-R (*R*=.89, *P*=.01).

**Conclusions:**

The mean quality of webpages with information for oral health care professionals about smoking cessation is low and displayed a high heterogeneity. These webpages are also difficult to read and often lack multimedia resources, which further limits their usefulness.

## Introduction

Oral health care professionals are well placed to motivate and dispense smoking cessation advice to their patients [1]. Tobacco plays a major role in the development and poor treatment outcomes of many oral diseases. The most serious consequence of tobacco use in the oral cavity is the increased risk of oral squamous cell carcinoma. There is a strong dose-response relationship between tobacco smoking and the development of potentially malignant disorders and oral cancer [2,3].

Tobacco use is also a risk factor for periodontal disease (including increased periodontal pockets depth; increased insertion loss, and as a consequence, dental mobility; increased tooth loss; gingival recessions; increased risk of failure of dental implants; increased risk of perimplantitis; and worse response to surgical and nonsurgical periodontal therapy) [4]. Tobacco has also been associated with delayed healing following oral surgery and an increased risk of alveolar osteitis following tooth extraction [5]. In addition, tobacco use has also been associated with halitosis, tooth and dental restorations staining, gingival pigmentation, and reduced taste sensation [6].

There is strong evidence that smoking cessation results in oral health benefits [7]. Smoking cessation is associated with the potential for reversal of premalignant oral disorders, enhanced outcomes following periodontal treatment, and better periodontal status compared with individuals who continue to smoke. The risk for oral cancer and periodontal disease progression of former smokers approximates to that of never smokers after 10 years of complete tobacco cessation [8].

To encourage oral health professionals to become more involved in smoking cessation, a care pathway based on recognized national and international guidelines has been produced by the European Workshop on Tobacco Use Prevention and Cessation for Oral Health Professionals. This is recommended as guidance for tobacco use cessation activity in dental practice. This guideline recommends an evidence-based technique called the “5As” approach: *A* sk about tobacco use, *A* dvise them to quit, *A* ssess willingness to quit, *A* ssist with quitting attempts, and *A* rrange for follow-up [9].

Research has confirmed that members of the dental team can be effective in assessing and advising tobacco users to quit [10]. Despite this, members of the dental team often cite issues such as lack of time or education as a reason to not offer smoking cessation advice to all smoking patients [11,12]. Support and training for oral health professionals can be provided through face-to-face contact but also via the Internet [13]. It has been shown that Web-based training for health care professionals, including dentists, can increase number of referrals to stop smoking services, and importantly, the rate of referrals converted to quit-line registrations. There is also evidence to suggest that training could improve provider knowledge, alongside improving attitudes toward tobacco cessation services, resulting in increased self-efﬁcacy for providing appropriate interventions [14]. Studies have been conducted regarding the content and quality of Web-based information among patients searching for smoking cessation advice [15-17]. However, there is no information regarding the quality of Web-based smoking cessation information for oral health care professionals.

The aim of this study was to identify and evaluate the quality of the content of webpages that provide information about smoking cessation for oral health care professionals.

## Methods

### Website Identification

Websites were identified on February 18, 2017 using Google and Health on Net (HON) medical professional search engines using the terms “smoking cessation OR quit smoking OR stop smoking OR 3As OR 5As OR tobacco counselling AND dentistry OR dental clinic OR dentist OR dental hygienist OR oral health professionals” written in English, without predetermined location or filters. The websites were displayed (10 sites per page), accessed, and saved for subsequent analysis.

The first 100 consecutive results from both search engines were considered for the study. Exclusion criteria were non-English language; irrelevant content; links to PubMed scientific articles; exclusively commercial information; patient-targeted sites; duplicated websites, forums, and discussion groups; non-operative sites; and password-protected webpages.

The review process was independently undertaken by 2 observers (AI and MD); in case of disagreement, a third reviewer (coordinator) was involved.

### Evaluation Procedures

The websites were grouped based on their affiliation (commercial, nonprofit, medical or university centers, government, professional societies) and level of specialization (exclusively dedicated to smoking cessation or partially dedicated to smoking cessation).

### Quality Assessment

Quality assessment was rated using the DISCERN questionnaire, the Journal of the American Medical Association (JAMA) benchmarks and the HON seal.

DISCERN is a validated questionnaire of 16 points, consisting of 8 questions examining reliability (questions 1-8) and specific details of information on treatment options (question 9-15) plus an overall quality score (question 16). Each question is classified in a numerical scale of 1 to 5 (1=very poor, 2=poor, 3=moderate, 4=good, 5=excellent). DISCERN has been designed to help users of consumer health information judge the quality of written information about treatment choices. Additionally, DISCERN has demonstrated interobserver reliability and construct validity when used by both medical and nonmedical professionals [18].

The JAMA benchmarks propose four basic standards of quality that include authorship of medical content (authors and contributors, relevant affiliations and credentials), attribution (list of references and sources of information), disclosure (website, sponsorship, advertising, commercial financing arrangements, conflicts of interest), and currency (content of the published and updated dates) [19].

Selected websites were also categorized by the presence of the HON seal. The HON seal is awarded to websites that meet with eight basic quality criteria: (1) authorship, (2) complementarity, (3) privacy, (4) attribution of references and currency, (5) justification, (6) transparency of the author, (7) sponsor transparency (financial disclosure), and (8) honesty in advertising policy [20].

### Smoking Cessation Content Assessment

The smoking cessation content on each site was assessed using an abbreviated version of the Smoke Treatment Scale (STS-C) and the Smoking Treatment Scale-Rating (STS-R) [17]. The STS-C is a 12-item checklist on which website reviewers documented the extent to which each website covered material related to key components of treatment as described in the US Public Health Service guidelines for the treatment of tobacco dependence. The resulting 12 items on the STS-C are as follows: (1-2) advise every smoker to quit smoking (subdivided into two categories: clear or strong and personalized), (3) assess readiness to quit, (4-5) assist with a quit plan (subdivided into three actions related to setting a quit date and seven topics for providing practical counseling), (6) provide intratreatment social support, (7) recommend use of approved pharmacotherapy, (8) arrange follow-up and four areas aimed at enhancing motivation to quit by discussing the (9) relevance of quitting smoking, (10) the risks of continued smoking, (11) the rewards of quitting, and (12) the potential roadblocks or barriers to quitting smoking [17].

STS-R was developed to provide numeric ratings of quality of coverage for each of the key components of treatment documented in the STS-C. Each website received ratings for (1) coverage, (2) accuracy, and (3) interactivity. Coverage ratings were used to indicate the relative depth and breadth of the information provided in each topic area. The ratings used a 5-point scale. If the treatment component was not mentioned, it received a rating of 1. If the topic was mentioned very briefly, it received a rating of 2. Key components covered briefly but with sufficient detail to be adequately helpful to smokers seeking to quit were given a rating of 3. Sites that provided more detail and more extensive information were given ratings of either 4 or 5 depending on the extent of the information provided [17].

### Readability Assessment

The Flesch Reading Ease (FRES) and the Flesch-Kinkaid Reading Grade Level (FKRGL) were used to assess legibility of the selected websites. A Web-based tool to calculate readability (Readability Formulas) was employed for this purpose. We used the following readability formulas:

FRES=206.835−(1.015×Average number of words per sentence)−(84.6×Average number of syllables per word); FKRGL=(0.39×Average number of words per sentence)+(11.8×Average number of syllables per word)−15.59 [21].

The FRES score was categorized as very difficult (college graduate level) (scores 0-29); difficult (30-49); fairly difficult (50-59); standard (easily understood by 13- to 15-year-old students) (60-69); fairly easy (70-79); easy (80-89); and very easy (90-100) [22]. Websites were also graded according to the FKRGL scale as easy (≤6th-grade level) or difficult (≥10th-grade level) to read [23]. Additionally, websites were also classified as multimedia and nonmultimedia and friendly or nonfriendly.

### Statistical Analysis

Statistical analysis was expressed using mean, minimum, and maximum values. Spearman correlation coefficients were calculated to examine the relationship between the DISCERN and STS-R mean values of each website. The significance level chosen for all statistical tests was *P* ≤.05. The analyses were performed using SPSS Statistics version 23 software package (IBM Corp, Armonk, NY, USA).

## Results

**Figure 1:**
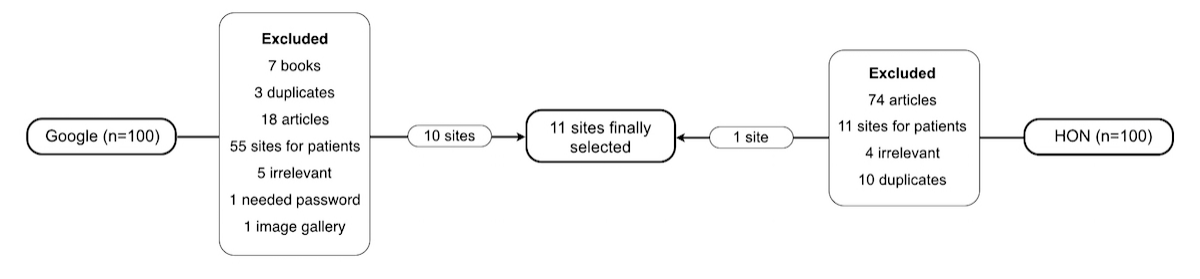
Schematic representation of the websites screening and the inclusion and exclusion process.

The search identified 1,680,000 sites on Google and 889,000 sites on the HON search engines. Of the first 200 sites selected (100 of Google and 100 of HON), only 11 met the inclusion criteria (Figure 1). The most common reasons for exclusion were scientific articles (92 out of 200), patient-specific sites (66 out of 200), and books (7 out of 200). Of the 11 websites analyzed, the majority belonged to governmental institutions (73%, 8/11), the others being prepared by Professional Associations (18%, 2/11) and commercial organizations (9%, 1/11). Only 27% (3/11) were exclusively dedicated to smoking cessation.

### Quality Assessment

**Figure 2:**
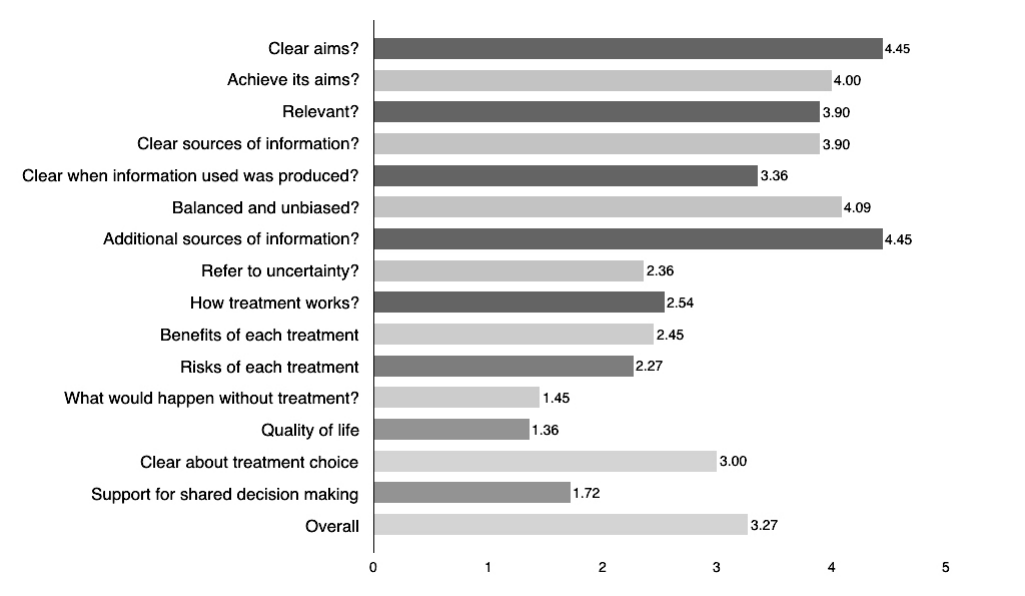
Median quality ratings scores of the 11 included websites using the DISCERN instrument.

The average score obtained with the DISCERN was 3.04 (standard deviation, SD 0.89). Mean quality ratings across the 11 included sites are shown in Figure 2. Mean score for the questions (1-8) that address reliability was 3.82 (SD 0.69) and for questions (9-15) that focus on specific details of the information about treatment choice was 2.26 (SD 0.69). The questions with the higher response score were as follows: “Does it provide details of additional sources of support or information?” and “Are the aims clear?” On the other hand, the question with the lowest score was “Does it describe how the treatment choices affect overall quality of life?

The results in relation to the JAMA benchmarks are shown in Table 1. None of the 11 evaluated websites achieved all four benchmarks, while 6 (54%), 2 (18%), 2 (18%), and 1 (9%) achieved 3, 2, 1, and 0 benchmarks, respectively. The highest scoring JAMA benchmark was authorship; over 80% identified the author. On the other hand, the lowest scoring benchmark was disclosure (9%) and this was usually because of the omission of financial details and conflicts of interest. None of the websites included in this study presented the HON seal.

**Table 1:** Website quality content based on Journal of the American Medical Association (JAMA) benchmarks.

JAMA benchmarks		n (%)
**Number of websites containing each benchmark**		
	4 benchmarks	0
	3 benchmarks	6 (54)
	2 benchmarks	2 (18)
	1 benchmarks	2 (18)
	0 benchmarks	1 (9)
**Percentage of included websites containing each benchmark**		
	Authorship	9 (82)
	Attribution	7 (64)
	Disclosure	1 (9)
	Currency	7 (64)

### Smoking Cessation Content

**Figure 3:**
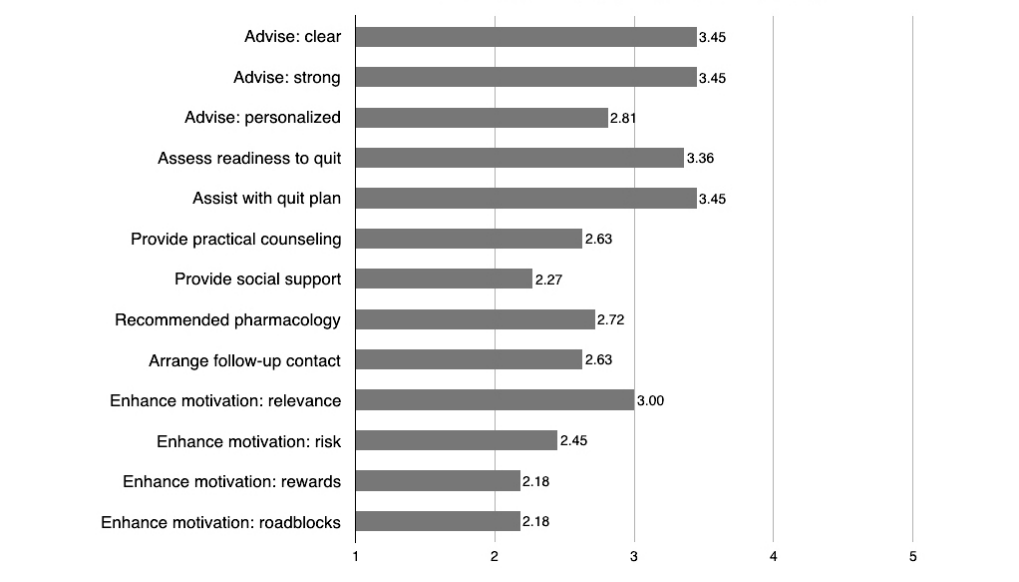
Median quality ratings scores of the 11 included websites using the Smoking Treatment Rating Scale (STS-R).

The results in relation to STS-C and STS-R evaluation tool are shown in Table 2 and Figure 3, respectively. All the sites contained a quit tobacco advice and a quit plan assistance. Three out of 11 (27%) provided intratreatment social support, and 72% (8/11) included the use of pharmacotherapy.

The mean of all parameters of STS-R was 2.81 (SD 0.95). The highest scores (3.45 [SD 0.82]) were obtained in clarity and strength advice and planning the quit. On the contrary, the lowest values were obtained in the rewards and roadblocks parameters (2.18 [SD 1.33]).

**Figure 4:**
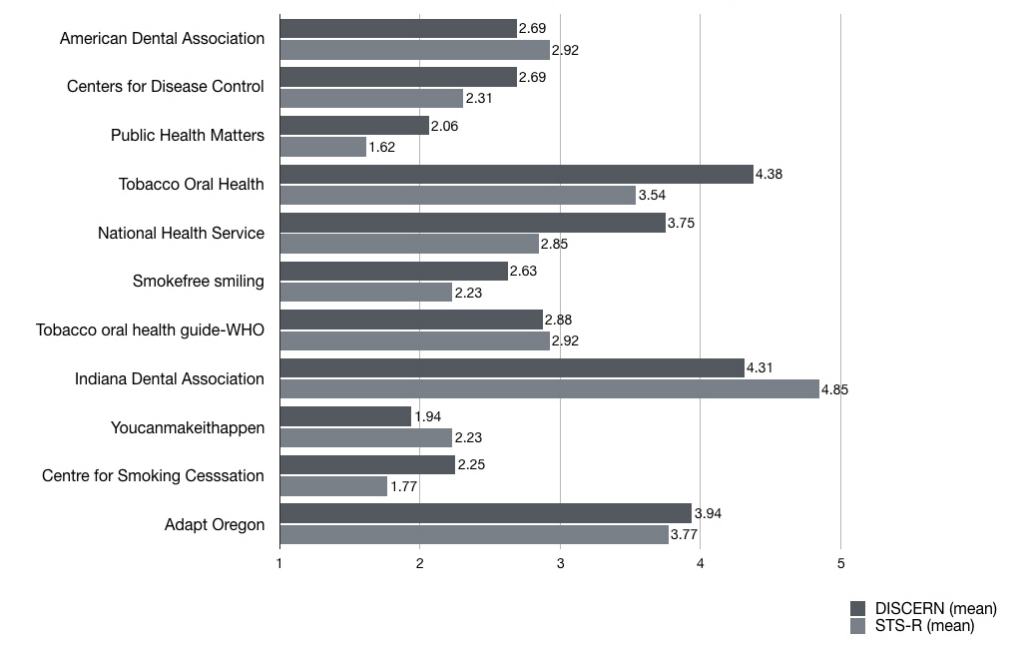
Correlation between DISCERN and Smoking Treatment Rating Scale (STS-R) grading scores. A significant positive correlation was obtained between the DISCERN mean values and the STS-R (R=.895, P=.01).

A significant strong positive correlation was obtained between the DISCERN mean values and the STS-R (*R*=.89, *P*=.01; Figure 4).

**Table 2:** Content analysis: Smoking Treatment Content Scale.

Smoking Treatment Content Scale (STS-C)	n (%)
Advise every tobacco user to quit	11 (100)
Assess readiness to quit	10 (91)
Assist with a quit plan	11 (100)
Provide practical counseling	6 (54)
Provide intratreatment social support	3 (28)
Recommend use of approved pharmacotherapy	8 (73)
Arrange follow-up contact	7 (64)
Enhance motivation: relevance	9 (82)
Enhance motivation: risks	6 (55)
Enhance motivation: rewards	5 (45)
Enhance motivation: roadblocks	4 (36)

**Table 3:** Features of the selected websites by content and quality rating.

Website name	Country	Affiliation	Site type	Flesch reading	Flesch-Kinkaid	JAMA^a^ benchmark	DISCERN mean	Smoking treatment rating scale (STS-R)
Indiana Dental Association	United States of America	Indiana Dental Association	Dental society	25.6	16	3	4.31	4.85
Adapt Oregon	United States of America	Private page	Commercial	46.7	10.2	2	3.94	3.77
Tobacco Oral Health	Switzerland	Oral Health Network on Tobacco Use Prevention and Cessation^b^	Governmental	40	13	3	4.38	3.54
American Dental Association	United States of America	American Dental Association	Dental society	17.3	16.4	1	2.69	2.92
Tobacco oral health guide	United States of America	World Health Organization	Governmental	32	13.9	3	2.88	2.92
National Health Service	United Kingdom	National Institute for Health Research	Governmental	43.7	11	3	3.75	2.85
Centers for Disease Control	United States of America	Centers for Disease Control and Prevention	Governmental	75.2	7	3	2.69	2.31
Smokefree smiling	United Kingdom	Government of the United Kingdom	Governmental	48.6	12.1	3	2.63	2.23
Youcanmakeithappen	Canada	Public Health Units of Canada	Governmental	35.2	17.7	0	1.94	2.23
Centre for Smoking Cessation	United Kingdom	The National Centre for Smoking Cessation and Training	Governmental	31.7	17.4	1	2.25	1.77
Publichealthmatters	United Kingdom	Government of the United Kingdom	Governmental	52.1	11.8	2	2.06	1.62

^a^JAMA: Journal of the American Medical Association.

^b^The Oral Health Network on Tobacco Use Prevention and Cessation (OHNTPC) is a subsidiary of the Swiss Task Force Tobacco-Interventions in dental practices.

### Readability Assessment

**Figure 5:**
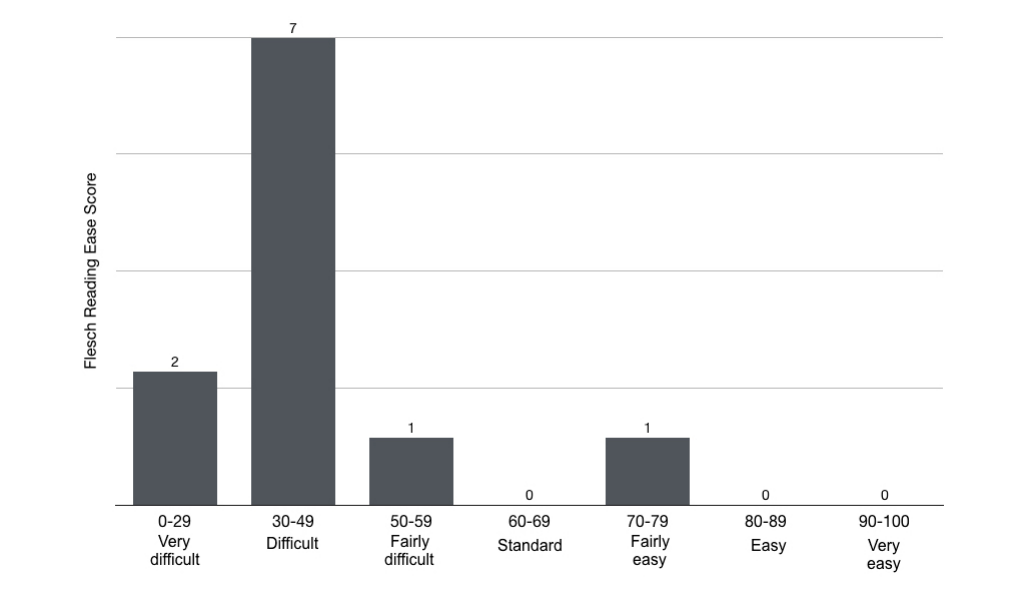
Frequency distribution of Flesch Reading Ease score of included websites.

Most of the assessed webpages 64% (7/11) showed a FRES of 30 to 49, and 82% (9/11) were scored between 0 and 49 points. One webpage obtained a score of 50 to 59 and another one 70 to 79 (Figure 5). The mean FRES was 40.73 (SD 15.46) and the mean FKRGL was 13.31 (SD 3.34).

Moreover, 45% (5/11) webpages showed their content in a PDF file. Just one of the webpages (9%) contained multimedia files and 45% (5/11) were considered as having a friendly usability.

Features of the 11 selected websites by content and quality rating are shown in Table 3.

## Discussion

### Principal Findings

The goal of this study was to assess and examine the content of webpages with information for oral health care professionals about smoking cessation. After applying the inclusion and exclusion criteria described, just 11 webpages with information on smoking cessation for oral health professionals were analyzed. Unfortunately, the main finding of our review was the small number of websites found in the search. In addition to the scarce number and low quality of content, the order of appearance might also affect the effectiveness of the search. In fact, the best 3 websites ranked by STS-R (Table 3) were found in the position 18th, 96th, and 47th, respectively. The results of a study by SISTRIX GmbH reported by AOL (America OnLine) in 2006 indicated that the chance of a site being accessed by a user, if ranked as the first result on an Internet search engine, was 59.6%. This reduced to 0.73% for the 10th place. The other combined 90 places (until reaching the 100th position) had a chance of 0.9%. On the basis of these data, a routine search might not be effective because of the browser algorithm, even if the website shows an adequate content.

As health professionals, dentists, dental hygienists, and dental assistants can play an important role in primary and secondary prevention of tobacco addiction. Brief tobacco dependence treatment provided by health care professionals, including dentists, is an effective way to prevent and reduce tobacco use [24].

Oral health professionals are in a unique position to motivate and assist their patients to quit smoking [1]. According to the latest meta-analysis performed by Carr and Ebbert in 2012, interventions for tobacco users delivered by oral health professionals can increase the odds of quitting tobacco (OR 2.38, 95% CI 1.70-3.35) [10,25]. Smoking cessation programs conducted through dental practices report cessation rates comparable with studies in other primary care settings [26]; however, we did not find studies comparing interventions conducted by oral health professionals and other health professionals.

Brief advice lasting less than 3 min given by a health professional will help an additional 2% of smokers to successfully stop smoking. With more intensive support lasting up to 10 min, plus nicotine replacement therapy, an additional 6% of the smokers will quit. By referring to stop smoking services, this increases by 15% to 20% [27,28].

Studies in private practice and dental schools ascertaining the knowledge and attitudes of dental health care professionals and students reveal that oral health professionals are aware of their responsibility to advise their patients to quit smoking. However, they do not feel sufficiently educated to help or advise their patients in a smoking cessation attempt. Therefore, smoking patients who seek help for smoking cessation are often assisted poorly from professionals within dentistry. It could be assumed that an improvement in the education of dentists and dental hygienists regarding interventions for smoking cessation could result in an increase in self-confidence and the frequency of their provision [29].

Although theoretical education about smoking is addressed in most European dental schools, more practical training in prevention and skills of implementing smoking cessation techniques are needed [30]. A recent survey reveals that although most dental schools in the United States and Canada provide tobacco dependence education, this is not a curricular component in all dental schools in the United States and Canada. The survey responses revealed that faculty members were most confident in teaching tobacco-related pathology but may lack the interest and skills needed to integrate tobacco dependence education as part of patient care [31].

These findings may partly explain the low level of adherence to tobacco use cessation guidelines among oral health professionals [32-35]. Effective tobacco cessation training should include skills and strategies that address student perceptions to foster the belief that tobacco cessation efforts are a part of quality clinical practice [36]. There is evidence that the training of health professionals in interventions for smoking cessation is associated with an increase in the smoking cessation rate [37].

Web-based education about the treatment of tobacco dependence could be an important way to build the understanding necessary to provide evidence-based treatment for tobacco dependence [38] and complement tobacco education received during undergraduate or postgraduate training. Houston et al demonstrated that a training program for oral health professionals, through a website designed to promote and support tobacco control in dental practice, can be effective. The intervention provided by a structured dynamic webpage increased the rates of detection of tobacco use and cessation advice for tobacco users. This result supports the potential of the Internet for oral health professional training in tobacco use cessation [39].

However, the Internet seems to be a relevant but underused tool to seek health information by health professionals, and one of the barriers described for its use by health professionals is that Web-based information is heterogeneous in quality [40].

The content and quality of health care information available on the Internet for patients searching for smoking cessation advice [15-17] and e-learning training programs about tobacco cessation for health care practitioners [38] have been reviewed in the literature. Selby et al reviewed and evaluated e-learning training programs about tobacco cessation for health care practitioners and found an overall poor quality of Web-based courses. Their results indicated that there is a widespread lack of well-designed Web-based continuing education courses in tobacco dependence treatment based on an analysis of instructional design quality [38].

However, no information about the quality of available Web-based smoking cessation (training/learning) for oral health professionals was reported.

The results of this study suggest that very few websites display high standards according to the DISCERN tool. DISCERN has been designed to help users of consumer health information judge the quality of written information about treatment choices. However, despite its potential interest, DISCERN is rarely used by patients and consumers in general [41]. Despite the lack of mainstream usage, it has been proven to be a reliable instrument when used by professionals with good interexaminer reliability [42]. Moreover, in this study, a significant strong positive correlation was obtained between the DISCERN mean values and the STS-R.

The JAMA benchmark is a condensed and relatively easy-to-apply tool to assess the reliability of health webpages and has been shown to correlate with high levels of accuracy [43,44].

In this study, of the websites that met the inclusion criteria, none displayed the HON seal. Although the HON seal indicates the reliability of a website, it does not imply that the reviewed websites lack reliability. As receipt of the HON seal must be requested, websites that do not display the HON seal may simply have not applied for, or are unaware of, the scheme. This does not mean that they do not adhere to the criteria proposed by the HON Foundation [45,46].

When applying the FRES tool to assess the readability of the selected webpages, it was found that most (81.8%) content was classified as “difficult” or very “difficult to read”. In the same way, the mean FKRGL (above 13th grade) showed that the assessed webpages were difficult to read. As the webpages were specific to dental practitioners, this is not as relevant as it would be in patient-centered websites. Regardless, clearer content should be advocated. Similarly, almost half of the websites presented their content in a PDF file, resulting in a more difficult way to access the text and read it. Just one of the sites included multimedia content with videos showing examples to the practitioners, advice, and tips to better explain the patients on how to quit tobacco use. Lack of multimedia content and a friendly graphic interface might limit the use of these sites.

With regard to the presence of contents using the STS-C, most of the websites (90-100%) included the advice on quitting tobacco, the readiness of the patient to quit, and the assistance of creating a plan to quit along the time. Recommendation of supplemental pharmacotherapy was included in 73% of the sites but just the 28% presented with information about the relevance of the social support or difficulties (roadblocks 36%) during the process. The quality of the Web content was higher in the Advise, Assess, and Assist phases (mean 3.45 [SD 0.82], 3.36 [SD 1.03], and 3.45 [SD 1.04], respectively). On the contrary, the websites failed in the personalization of the message (mean 2.81 [SD 1.17]), highlighting to the dentist the need to understand the specific situation of each patient and modulate the message to them. As stated before, the social support was ranked inferiorly (mean 2.27 [SD 1.10]) and so were the presence of practical counseling (mean 2.63 [SD 0.92]) and the presence of rewards and roadblocks (mean 2.18 [SD 1.33]).

### Limitations

Some limitations of this study should be highlighted. This study cannot be considered an exhaustive analysis since only webpages written in English were revised. In addition, only webpages addressed to oral health professionals were considered. For this reason, it is possible that webpages that were not directly addressed to oral health professionals but which may contain useful information and could be equally applied in the dental setting could have been excluded. Therefore, generalization of the overall context of results is limited, and similar reviews should be considered on websites not written in English and addressed to other health professionals.

### Future Work

After assessing the quality of the content available on webpages with information for oral health care professionals about smoking cessation, shortcomings in the available educational resources were identified. Developing of e-learning materials on the topic to improve the skills, self-confidence, and frequency of provision of interventions for smoking cessation in the dental setting by members of the dental team is encouraged.

There have been recommendations for the development of dental “continuing professional development” e-learning resources. Such resources must be learner-friendly, interactive, and allow the user to gain knowledge at a rate that is appropriate to the individual. There should also be flexibility, alongside the opportunity to critically analyze data either individually or as part of a team. Content should be relevant, accurate, easy to access, and regularly evaluated and updated when necessary. The visual design of the module’s webpage should be attractive, appropriate, and uncomplicated, with content presented in a manner to facilitate easy reading and to guide the learner appropriately through the content. Feedback should be available for those who use the resource. Colors, graphics, animations, and different media should be used to complement or provide information in an educationally useful manner [47].

On the basis of a European Union (EU) initiative for lifelong learning, our group has been commissioned to deliver a Web-based learning program designed to be used by health care professionals, including dentists and dental hygienists, to increase their professional skills in providing smoking cessation advice for tobacco users. This can be accessed online [48].

To assess the utility of this resource, we aim to (1) carry out an evaluation of the webpage by external experts; (2) subsequently extend the evaluation to health care professionals, including dentists and oral hygienists from different countries, translating the text, and adapting content to incorporate local policy; and (3) finally investigate whether the resource has caused a change in the user’s routine clinical practice via feedback questionnaires.

### Conclusions

In conclusion, the number of smoking cessation webpages for oral health care professionals is scarce and displayed a low quality and high heterogeneity in their content. We found it difficult to find good quality information, an absence of multimedia resources and readability levels, which further limited the usefulness of most websites.

## Abbreviations

EU: European Union
FKRGL: Flesch-Kinkaid Reading Grade Level
FRES: Flesch Reading Ease
HON: Health on Net
JAMA: Journal of the American Medical Association
SD: standard deviation
STS-C: Smoke Treatment Scale
STS-R: Smoking Treatment Scale-Rating
